# Crystal structure of (Na_0.70_)(Na_0.70_,Mn_0.30_)(Fe^3+^,Fe^2+^)_2_Fe^2+^(VO_4_)_3_, a sodium-, iron- and manganese-based vanadate with the alluaudite-type structure

**DOI:** 10.1107/S2056989016000931

**Published:** 2016-01-23

**Authors:** Elhassan Benhsina, Abderrazzak Assani, Mohamed Saadi, Lahcen El Ammari

**Affiliations:** a Laboratoire de Chimie du Solide Appliquée, Faculté des Sciences, Université Mohammed V de Rabat, Avenue Ibn Battouta, BP 1014, Rabat, Morocco

**Keywords:** crystal structure, transition metal vanadate, solid-state reaction synthesis, alluaudite-type structure

## Abstract

The title compound, sodium (sodium,manganese) triiron(II,III) tris[vana­date(V)], (Na_0.70_)(Na_0.70_,Mn_0.30_)(Fe^3+^,Fe^2+^)_2_Fe^2+^(VO_4_)_3_, was prepared by solid-state reactions. It crystallizes in an alluaudite-like structure, characterized by a partial cationic disorder. In the structure, four of the 12 sites in the asymmetric unit are located on special positions, three on a twofold rotation axis (Wyckoff position 4*e*) and one on an inversion centre (4*b*). Two sites on the twofold rotation axis are entirely filled by Fe^2+^ and V^5+^, whereas the third site has a partial occupancy of 70% by Na^+^. The site on the inversion centre is occupied by Na^+^ and Mn^2+^ cations in a 0.7:0.3 ratio. The remaining Fe^2+^ and Fe^3+^ atoms are statistically distributed on a general position. The three-dimensional framework of this structure is made up of kinked chains of edge-sharing [FeO_6_] octa­hedra stacked parallel to [10-1]. These chains are held together by VO_4_ tetra­hedral groups, forming polyhedral sheets perpendicular to [010]. Within this framework, two types of channels extending along [001] are present. One is occupied by (Na^+^/Mn^2+^) while the second is partially occupied by Na^+^. The mixed site containing (Na^+^/Mn^2+^) has an octa­hedral coordination sphere, while the Na^+^ cations in the second channel are coordinated by eight O atoms.

## Abstract 2

The title transition metal vanadate crystallizes in an alluaudite-type structure. The chains characterizing the alluaudite structure are built up from edge-sharing [FeO_6_] octa­hedra linked together by VO_4_ tetra­hedra.

## Chemical context   

Over recent decades, the synthesis and structural characterization of transition-metal-based functional materials adopting layered or channel structures has been the focus of much scientific work. In accordance with widespread studies devoted to the improvement of those materials, we have contributed to the search for new functional materials by undertaking synthesis and structural characterization of new transition and alkali metal phosphates exhibiting channel structures and belonging to the well-known alluaudite structure type (Moore, 1971 ▸) that can be represented by the general formula *A*(1)*A*(2)*M*(1)*M*(2)_2_(*X*O_4_)_3_. The *M*(1) and *M*(2) sites accommodate di- or trivalent cations in an octa­hedral environment and are connected to the tetra­hedral *X*O_4_ groups, leading to an open-framework structure. Alluaudite-type phosphates are of special inter­est as positive electrode materials in lithium and sodium batteries. For instance, the alluaudite-type lithium manganese phosphate Li_0.78_Na_0.22_MnPO_4_ is proposed by Kim *et al.* (2014 ▸) as a promising new positive electrode for Li rechargeable batteries. Furthermore, in the more active alluaudite-type cathode material for sodium-ion batteries, Na_2_Fe_3-*x*_Mn_*x*_(PO_4_)_3_, the electrochemical performance is associated either with morphology or with the electronic and crystalline structure (Huang *et al.*, 2015 ▸).

Responding to the growing demand for this type of functional materials, we were able to prepare new alluaudite-type phosphates within pseudo-ternary *A*_2_O/*M*O/P_2_O_5_ or pseudo-quaternary *A*_2_O/*M*O/Fe_2_O_3_/P_2_O_5_ systems by means of hydro­thermal or solid-state reactions: AgMg_3_(HPO_4_)_2_PO_4_ (Assani *et al.*, 2011 ▸), NaMg_3_(HPO_4_)_2_PO_4_ (Ould Saleck *et al.*, 2015 ▸), Na_2_Co_2_Fe(PO_4_)_3_ (Bouraima *et al.*, 2015 ▸) and Na_1.67_Zn_1.67_Fe_1.33_(PO_4_)_3_ (Khmiyas *et al.*, 2015 ▸).

Besides well-known phosphate phases, arsenates (Đorđević *et al.*, 2015 ▸; Stock & Bein, 2003 ▸) and more recently molybdates (Nasri *et al.*, 2014 ▸; Savina *et al.*, 2014 ▸) and sulfates (Oyama *et al.*, 2015 ▸; Ming *et al.*, 2015 ▸) have been reported to crystallize with alluaudite-type structures. However, to the best of our knowledge, no vanadate adopting this type of structure has been reported so far. Therefore we performed hydro­thermal and solid-state reaction investigations within the *A*_2_O/*M*O/*M*′_2_O_3_/V_2_O_5_ system (*A* = monovalent cation, *M* = bivalent cation and *M*′ = trivalent cation) with approximate molar ratios of *A*:*M*:*M*′:V = 2:2:1:3 and report here details of the preparation and structural characterization of the first sodium- manganese- and iron-based vanadate with an alluaudite-type structure, *viz*. (Na_0.70_)(Na_0.70_,Mn_0.30_)(Fe^3+^,Fe^2+^)_2_Fe^2+^(VO_4_)_3_.

## Structural commentary   

**Figure 1:**
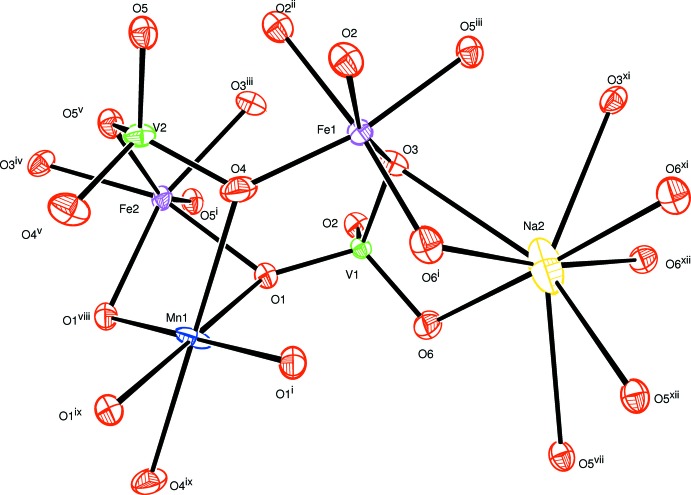
The principal building units in the structure of the title compound. Displacement ellipsoids are drawn at the 50% probability level. [Symmetry codes: (i) *x*, −*y* + 1, *z* + 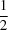; (ii) *x*, *y*, *z* + 1; (iii) −*x* + 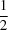, −*y* + 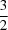, −*z* + 2; (iv) −*x* + 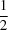, −*y* + 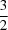, −*z* + 1; (v) −*x*, *y*, −*z* + 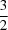; (vi) *x*, *y*, *z* − 1; (vii) *x* − 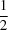, −*y* + 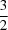, *z* − 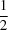; (viii) −*x*, *y*, −*z* + 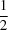; (ix) −*x*, −*y* + 1, −*z* + 1; (*x*) *x*, −*y* + 1, *z* − 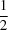; (xi) −*x* + 1, *y*, −*z* + 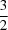; (xii) −*x* + 1, −*y* + 1, −*z* + 1.]

The preparation of this compound by melting a mixture of three metal oxide precursors in addition to vanadium oxide forced us to explore several crystallographic models. Refinement of the occupancy ratios, bond-valence analysis and the electrical neutrality requirement of the structure lead to the given composition for the title compound. The basic building units of the structure are shown in Fig. 1 ▸. The structure is characterized by disorder in three positions. Fe1^2+^ and Fe1^3+^ are statistically distributed on a general site (Wyckoff position 8*f*); Na1^+^ and Mn1^2+^ are disordered in a 0.7:0.3 ratio on a site located on an inversion centre (4*b*), and Na2^+^ is present at a site on a twofold rotation axis (4*e*) with 70% occupancy. All other sites are fully occupied. Nearly the same cationic distribution was reported by Yakubovich *et al.* (1977 ▸) for the alluaudite-type phosphate Na_2_(Fe^3+^,Fe^2+^)_2_Fe^2+^(PO_4_)_3_.

**Figure 2:**
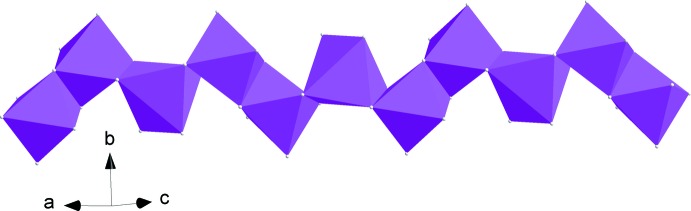
Edge-sharing [FeO_6_] octa­hedra forming a kinked chain running parallel to [10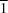].

**Figure 3:**
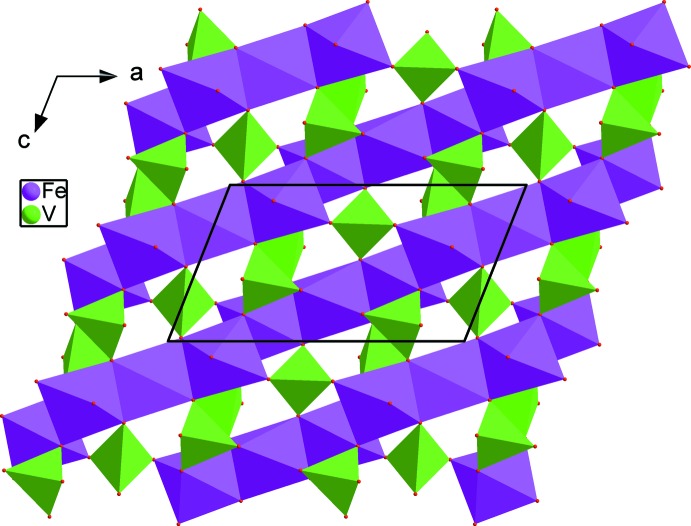
A layer perpendicular to [010], resulting from the connection of chains *via* vertices of VO_4_ tetra­hedra.

**Figure 4:**
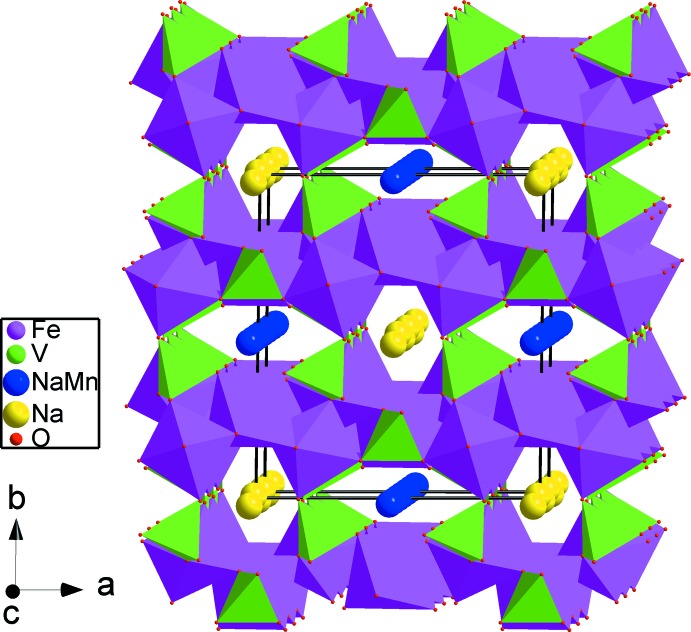
Polyhedral representation of (Na_0.70_)(Na_0.70_Mn_0.30_)(Fe^3+^/Fe^2+^)_2_Fe^2+^(VO_4_)_3_, showing channels running along and parallel to [001].

The crystal structure of the title compound is built up from edge-sharing [FeO_6_] octa­hedra, leading to the formation of kinked chains running along [10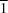] (Fig. 2 ▸). These chains are held together through the vertices of VO_4_ tetra­hedra, generating layers perpendicular to [010] (Fig. 3 ▸). Thereby an open three-dimensional framework is formed that delimits two types of channels parallel to [001] in which the disordered (Na1^+^/Mn1^2+^) and statistically occupied Na2^+^ cations are accommodated (Fig. 4 ▸). The (Na1^+^,Mn1^2+^) site has a distorted octa­hedral oxygen environment, with (Na1^+^,Mn1^2+^)—O bond lengths between 2.4181 (16) and 2.5115 (15) Å. The Na2^+^ cation is coordinated by eight oxygen atoms with Na2—O distances in the range 2.4879 (18) to 2.982 (3) Å. The disorder of Na^+^ in the channels might admit ionic mobility for this material.

## Synthesis and crystallization   

The title compound was prepared by solid-state reactions in air. Sodium nitrate, metallic manganese and iron were mixed with vanadium oxide in proportions corresponding to the molar ratios Na:Mn:Fe:V = 2:2:1:3. The reaction mixture underwent several heat treatments in a platinum crucible until the melting temperature situated at about 1030 K was reached. Each thermal treatment was inter­spersed with grinding in an agate mortar. The resulting product contained black single crystals crystals of a suitable size for the X-ray diffraction study.

## Refinement   

Crystal data, data collection and structure refinement details are summarized in Table 1 ▸. For the (Na1^+^,Mn1^2+^) site, full occupation was assumed, with the sum of the site occupation factors constrained to be 1. The site-occupation factor of Na2^+^ was refined freely. In the last step of the refinement, the site occupation factors were fixed to fulfill electro-neutrality. Reflection (1 5 0) was probably affected by the beam-stop and was omitted from the refinement. The remaining maximum and minimum electron density peaks are located 0.59 and 0.41 Å from Fe2 and V2, respectively.

## Supplementary Material

Crystal structure: contains datablock(s) I. DOI: 10.1107/S2056989016000931/wm5259sup1.cif

Structure factors: contains datablock(s) I. DOI: 10.1107/S2056989016000931/wm5259Isup2.hkl

CCDC reference: 1447912

Additional supporting information:  crystallographic information; 3D view; checkCIF report

## Figures and Tables

**Table 1:** Experimental details

Crystal data	
Chemical formula	Na_1.40_Mn_0.30_Fe_3_(VO_4_)_3_
*M*_r_	561.04
Crystal system, space group	Monoclinic, *C*2/*c*
Temperature (K)	296
*a*, *b*, *c* (Å)	11.9512 (5), 12.9022 (5), 6.7756 (3)
β (°)	111.678 (1)
*V* (Å^3^)	970.88 (7)
*Z*	4
Radiation type	Mo *K*α
μ (mm^−1^)	7.63
Crystal size (mm)	0.30 × 0.26 × 0.18

Data collection	
Diffractometer	Bruker X8 APEX
Absorption correction	Multi-scan (*SADABS*; Bruker, 2009 ▸)
*T*_min_, *T*_max_	0.545, 0.747
No. of measured, independent and observed [*I* > 2σ(*I*)] reflections	17759, 1768, 1595
*R*_int_	0.030
(sin θ/λ)_max_ (Å^−1^)	0.757

Refinement	
*R*[*F* ^2^ > 2σ(*F* ^2^)], *wR*(*F* ^2^), *S*	0.020, 0.056, 1.12
No. of reflections	1768
No. of parameters	100
Δρ_max_, Δρ_min_ (e Å^−3^)	0.74, −0.99

Computer programs: *APEX2* and *SAINT* (Bruker, 2009 ▸), *SHELXT* (Sheldrick, 2015*a* ▸), *SHELXL2014* (Sheldrick, 2015*b* ▸), *ORTEP-3 for Windows* (Farrugia, 2012 ▸), *DIAMOND* (Brandenburg, 2006 ▸) and *publCIF* (Westrip, 2010 ▸).

## supplementary crystallographic information

### Crystal data

**Table:**

Na_1.40_Mn_0.30_Fe_3_(VO_4_)_3_	*F*(000) = 1064
*M**_r_* = 561.04	*D*_x_ = 3.838 Mg m^−^^3^
Monoclinic, *C*2/*c*	Mo *K*α radiation, λ = 0.71073 Å
*a* = 11.9512 (5) Å	Cell parameters from 1768 reflections
*b* = 12.9022 (5) Å	θ = 2.4–32.6°
*c* = 6.7756 (3) Å	µ = 7.63 mm^−^^1^
β = 111.678 (1)°	*T* = 296 K
*V* = 970.88 (7) Å^3^	Block, black
*Z* = 4	0.30 × 0.26 × 0.18 mm

### Data collection

**Table:**

Bruker X8 APEX diffractometer	1768 independent reflections
Radiation source: fine-focus sealed tube	1595 reflections with *I* > 2σ(*I*)
Graphite monochromator	*R*_int_ = 0.030
φ and ω scans	θ_max_ = 32.6°, θ_min_ = 2.4°
Absorption correction: multi-scan (*SADABS*; Bruker, 2009)	*h* = −18→18
*T*_min_ = 0.545, *T*_max_ = 0.747	*k* = −19→19
17759 measured reflections	*l* = −7→10

### Refinement

**Table:**

Refinement on *F*^2^	100 parameters
Least-squares matrix: full	0 restraints
*R*[*F*^2^ > 2σ(*F*^2^)] = 0.020	*w* = 1/[σ^2^(*F*_o_^2^) + (0.0252*P*)^2^ + 2.9028*P*] where *P* = (*F*_o_^2^ + 2*F*_c_^2^)/3
*wR*(*F*^2^) = 0.056	(Δ/σ)_max_ = 0.001
*S* = 1.12	Δρ_max_ = 0.74 e Å^−^^3^
1768 reflections	Δρ_min_ = −0.99 e Å^−^^3^

### Special details

**Table:**

Geometry. All e.s.d.'s (except the e.s.d. in the dihedral angle between two l.s. planes) are estimated using the full covariance matrix. The cell e.s.d.'s are taken into account individually in the estimation of e.s.d.'s in distances, angles and torsion angles; correlations between e.s.d.'s in cell parameters are only used when they are defined by crystal symmetry. An approximate (isotropic) treatment of cell e.s.d.'s is used for estimating e.s.d.'s involving l.s. planes.

### Fractional atomic coordinates and isotropic or equivalent isotropic displacement parameters (Å^2^)

**Table:**

	*x*	*y*	*z*	*U*_iso_*/*U*_eq_	Occ. (<1)
Fe1	0.28812 (3)	0.65986 (2)	0.87842 (4)	0.00988 (7)	
Fe2	0.0000	0.73519 (3)	0.2500	0.01105 (9)	
V1	0.26720 (3)	0.61038 (2)	0.37946 (5)	0.00884 (7)	
V2	0.0000	0.71081 (4)	0.7500	0.01126 (10)	
Mn1	0.0000	0.5000	0.5000	0.0136 (8)	0.3
Na1	0.0000	0.5000	0.5000	0.0420 (18)	0.7
Na2	0.5000	0.4890 (3)	0.7500	0.0432 (7)	0.7
O1	0.12025 (14)	0.59837 (11)	0.3264 (3)	0.0151 (3)	
O2	0.28070 (14)	0.68158 (12)	0.1709 (2)	0.0160 (3)	
O3	0.33564 (14)	0.67141 (12)	0.6228 (2)	0.0148 (3)	
O4	0.11010 (16)	0.62915 (12)	0.7570 (3)	0.0194 (3)	
O5	0.03980 (14)	0.78286 (12)	0.9783 (2)	0.0138 (3)	
O6	0.33208 (16)	0.49277 (13)	0.3977 (3)	0.0189 (3)	

### Atomic displacement parameters (Å^2^)

**Table:**

	*U*^11^	*U*^22^	*U*^33^	*U*^12^	*U*^13^	*U*^23^
Fe1	0.01049 (13)	0.01185 (12)	0.00849 (13)	−0.00026 (9)	0.00491 (10)	−0.00052 (9)
Fe2	0.00964 (17)	0.01269 (17)	0.01294 (19)	0.000	0.00665 (14)	0.000
V1	0.00876 (14)	0.01060 (14)	0.00697 (15)	−0.00061 (10)	0.00267 (11)	−0.00072 (10)
V2	0.0160 (2)	0.00896 (18)	0.0073 (2)	0.000	0.00244 (16)	0.000
Mn1	0.0196 (17)	0.0093 (18)	0.0073 (18)	−0.0076 (13)	−0.0005 (14)	0.0011 (13)
Na1	0.059 (4)	0.027 (4)	0.033 (4)	−0.001 (3)	0.009 (3)	−0.001 (3)
Na2	0.0216 (12)	0.0687 (19)	0.0345 (14)	0.000	0.0047 (10)	0.000
O1	0.0108 (6)	0.0137 (6)	0.0201 (8)	−0.0008 (5)	0.0049 (6)	−0.0013 (5)
O2	0.0160 (7)	0.0203 (7)	0.0119 (7)	−0.0020 (5)	0.0053 (6)	−0.0005 (5)
O3	0.0176 (7)	0.0142 (6)	0.0117 (7)	−0.0049 (5)	0.0043 (5)	−0.0007 (5)
O4	0.0234 (8)	0.0136 (6)	0.0163 (7)	0.0027 (6)	0.0015 (6)	−0.0045 (5)
O5	0.0116 (6)	0.0177 (6)	0.0124 (7)	0.0003 (5)	0.0048 (5)	0.0003 (5)
O6	0.0173 (7)	0.0200 (7)	0.0191 (8)	0.0024 (6)	0.0063 (6)	−0.0056 (6)

### Geometric parameters (Å, º)

**Table:**

Fe1—O4	2.0167 (18)	Mn1—O4^ix^	2.4181 (16)
Fe1—O3	2.0180 (16)	Mn1—O4	2.4181 (16)
Fe1—O6^i^	2.0299 (17)	Mn1—O1^i^	2.4941 (16)
Fe1—O2^ii^	2.0358 (16)	Mn1—O1^viii^	2.4941 (16)
Fe1—O5^iii^	2.0599 (16)	Mn1—O1	2.5115 (15)
Fe1—O2^iv^	2.1841 (16)	Mn1—O1^ix^	2.5115 (15)
Fe2—O5^v^	2.1540 (15)	Na1—O4^ix^	2.4181 (16)
Fe2—O5^vi^	2.1540 (15)	Na1—O4	2.4181 (16)
Fe2—O3^iv^	2.1915 (15)	Na1—O1^i^	2.4941 (16)
Fe2—O3^vii^	2.1915 (15)	Na1—O1^viii^	2.4941 (16)
Fe2—O1^viii^	2.2136 (15)	Na1—O1	2.5115 (15)
Fe2—O1	2.2136 (15)	Na1—O1^ix^	2.5115 (15)
V1—O1	1.6647 (15)	Na1—O4^x^	2.9698 (18)
V1—O6	1.6878 (16)	Na1—O4^v^	2.9698 (18)
V1—O3	1.7351 (16)	Na2—O6^xi^	2.4879 (18)
V1—O2	1.7420 (16)	Na2—O6	2.4879 (18)
V2—O4	1.6726 (17)	Na2—O6^xii^	2.5627 (18)
V2—O4^v^	1.6726 (17)	Na2—O6^i^	2.5627 (18)
V2—O5	1.7147 (15)	Na2—O3^xi^	2.982 (3)
V2—O5^v^	1.7147 (15)	Na2—O3	2.982 (3)
			
O4—Fe1—O3	104.67 (7)	O1^i^—Mn1—O1	115.50 (6)
O4—Fe1—O6^i^	92.56 (7)	O1^viii^—Mn1—O1	64.50 (6)
O3—Fe1—O6^i^	88.77 (7)	O4^ix^—Mn1—O1^ix^	74.67 (6)
O4—Fe1—O2^ii^	90.31 (7)	O4—Mn1—O1^ix^	105.33 (6)
O3—Fe1—O2^ii^	162.33 (6)	O1^i^—Mn1—O1^ix^	64.50 (6)
O6^i^—Fe1—O2^ii^	100.04 (7)	O1^viii^—Mn1—O1^ix^	115.50 (6)
O4—Fe1—O5^iii^	169.09 (6)	O1—Mn1—O1^ix^	180.00 (6)
O3—Fe1—O5^iii^	80.18 (6)	O4^ix^—Na1—O4	180.0
O6^i^—Fe1—O5^iii^	97.36 (7)	O4^ix^—Na1—O1^i^	105.66 (5)
O2^ii^—Fe1—O5^iii^	83.50 (6)	O4—Na1—O1^i^	74.34 (5)
O4—Fe1—O2^iv^	80.83 (6)	O4^ix^—Na1—O1^viii^	74.34 (5)
O3—Fe1—O2^iv^	90.51 (6)	O4—Na1—O1^viii^	105.66 (5)
O6^i^—Fe1—O2^iv^	172.94 (7)	O1^i^—Na1—O1^viii^	180.0
O2^ii^—Fe1—O2^iv^	82.57 (6)	O4^ix^—Na1—O1	105.33 (6)
O5^iii^—Fe1—O2^iv^	89.43 (6)	O4—Na1—O1	74.67 (6)
O5^v^—Fe2—O5^vi^	146.82 (8)	O1^i^—Na1—O1	115.50 (6)
O5^v^—Fe2—O3^iv^	87.45 (6)	O1^viii^—Na1—O1	64.50 (6)
O5^vi^—Fe2—O3^iv^	74.36 (6)	O4^ix^—Na1—O1^ix^	74.67 (6)
O5^v^—Fe2—O3^vii^	74.36 (6)	O4—Na1—O1^ix^	105.33 (6)
O5^vi^—Fe2—O3^vii^	87.45 (6)	O1^i^—Na1—O1^ix^	64.50 (6)
O3^iv^—Fe2—O3^vii^	113.28 (8)	O1^viii^—Na1—O1^ix^	115.50 (6)
O5^v^—Fe2—O1^viii^	95.65 (6)	O1—Na1—O1^ix^	180.00 (6)
O5^vi^—Fe2—O1^viii^	110.91 (6)	O4^ix^—Na1—O4^x^	56.55 (7)
O3^iv^—Fe2—O1^viii^	160.16 (6)	O4—Na1—O4^x^	123.45 (7)
O3^vii^—Fe2—O1^viii^	86.35 (6)	O1^i^—Na1—O4^x^	88.74 (5)
O5^v^—Fe2—O1	110.91 (6)	O1^viii^—Na1—O4^x^	91.26 (5)
O5^vi^—Fe2—O1	95.65 (6)	O1—Na1—O4^x^	64.95 (5)
O3^iv^—Fe2—O1	86.35 (6)	O1^ix^—Na1—O4^x^	115.05 (5)
O3^vii^—Fe2—O1	160.16 (6)	O4^ix^—Na1—O4^v^	123.45 (7)
O1^viii^—Fe2—O1	74.22 (8)	O4—Na1—O4^v^	56.55 (7)
O1—V1—O6	110.59 (8)	O1^i^—Na1—O4^v^	91.26 (5)
O1—V1—O3	109.68 (8)	O1^viii^—Na1—O4^v^	88.74 (5)
O6—V1—O3	107.22 (8)	O1—Na1—O4^v^	115.05 (5)
O1—V1—O2	106.29 (8)	O1^ix^—Na1—O4^v^	64.95 (5)
O6—V1—O2	110.84 (8)	O4^x^—Na1—O4^v^	180.0
O3—V1—O2	112.27 (7)	O6^xi^—Na2—O6	177.75 (17)
O4—V2—O4^v^	101.92 (12)	O6^xi^—Na2—O6^xii^	84.40 (5)
O4—V2—O5	111.28 (8)	O6—Na2—O6^xii^	95.40 (5)
O4^v^—V2—O5	108.67 (8)	O6^xi^—Na2—O6^i^	95.40 (5)
O4—V2—O5^v^	108.67 (8)	O6—Na2—O6^i^	84.40 (5)
O4^v^—V2—O5^v^	111.28 (8)	O6^xii^—Na2—O6^i^	169.46 (16)
O5—V2—O5^v^	114.33 (10)	O6^xi^—Na2—O3^xi^	59.69 (6)
O4^ix^—Mn1—O4	180.0	O6—Na2—O3^xi^	118.28 (11)
O4^ix^—Mn1—O1^i^	105.66 (5)	O6^xii^—Na2—O3^xi^	60.86 (6)
O4—Mn1—O1^i^	74.34 (5)	O6^i^—Na2—O3^xi^	109.99 (10)
O4^ix^—Mn1—O1^viii^	74.34 (5)	O6^xi^—Na2—O3	118.28 (11)
O4—Mn1—O1^viii^	105.66 (5)	O6—Na2—O3	59.69 (6)
O1^i^—Mn1—O1^viii^	180.0	O6^xii^—Na2—O3	109.99 (10)
O4^ix^—Mn1—O1	105.33 (6)	O6^i^—Na2—O3	60.86 (6)
O4—Mn1—O1	74.67 (6)	O3^xi^—Na2—O3	75.74 (10)

Symmetry codes: (i) *x*, −*y*+1, *z*+1/2; (ii) *x*, *y*, *z*+1; (iii) −*x*+1/2, −*y*+3/2, −*z*+2; (iv) −*x*+1/2, −*y*+3/2, −*z*+1; (v) −*x*, *y*, −*z*+3/2; (vi) *x*, *y*, *z*−1; (vii) *x*−1/2, −*y*+3/2, *z*−1/2; (viii) −*x*, *y*, −*z*+1/2; (ix) −*x*, −*y*+1, −*z*+1; (x) *x*, −*y*+1, *z*−1/2; (xi) −*x*+1, *y*, −*z*+3/2; (xii) −*x*+1, −*y*+1, −*z*+1.
